# Seroprevalence of varicella-zoster virus and predictors for seronegativity in the Amsterdam adult population

**DOI:** 10.1186/1471-2334-12-140

**Published:** 2012-06-21

**Authors:** Gini GC van Rijckevorsel, Marjolein Damen, Gerard J Sonder, Maarten F Schim van der Loeff, Anneke van den Hoek

**Affiliations:** 1 Public Health Service Amsterdam, Department of Infectious Diseases, Nieuwe Achtergracht 100, 1018 WT, Amsterdam, the Netherlands; 2 Public Health Laboratory, Cluster of Infectious Diseases, Public Health Service of Amsterdam, Nieuwe Achtergracht 100, 1018 WT, Amsterdam, the Netherlands; 3 Academic Medical Centre, Department of Internal Medicine, Division of Infectious Diseases, Tropical Medicine and AIDS, Meibergdreef 9, 1105 AZ, Amsterdam, the Netherlands; 4 Public Health Service Amsterdam, Cluster Infectious Diseases, Department of Research, Nieuwe Achtergracht 100, 1018 WT, Amsterdam, the Netherlands

## Abstract

**Background:**

In the Netherlands, infection with varicella-zoster virus (VZV) is considered a benign common childhood illness and routine vaccination against VZV is not done. In 1995 it was estimated that 98-100% of the adult Dutch general population is immune, yet the estimate is based on a database in which a relative small number of people of non-Dutch ethnic origin were represented. As the city of Amsterdam has large immigrant communities originating from various subtropical and tropical countries, such as Morocco, Surinam, and Turkey with probably lower VZV transmission, this study aimed to estimate the seroprevalence of VZV IgG antibodies (anti-VZV) among various ethnic groups in Amsterdam, and identify factors associated with seronegative VZV status.

**Methods:**

The study was a cross-sectional survey of the Amsterdam population (2004), and the study sample was stratified by age and ethnicity, with deliberate oversampling of minority ethnic groups. Serum samples obtained from 1,341 residents in 2004 were tested for antibodies to VZV. Basic demographic data (gender, age, country of birth, age at immigration and number of children) were also available.

**Results:**

The anti-VZV seroprevalence in the overall Amsterdam population was estimated to be 94% (95% confidence intervals; 92–96%). Regarding ethnic origin, first generation immigrants (Moroccan immigrants 90%, Surinamese or Antillean immigrants 91%, and Turkish 92%), especially those that migrated after the age of 11 years, were more likely to be anti-VZV seronegative compared to those arriving at an earlier age or those born in the Netherlands (97–98%). Both ethnic origin and generation of immigration were positive predictors for IgG seronegativity to VZV (p<0.015). No other predictors for seronegativity were found.

**Conclusion:**

The results of this study imply that about 4–8% of the general adult Amsterdam population is still susceptible to infection with VZV, and that susceptibility is even higher in some immigrant groups. When assessing the risk of infection after VZV exposure alertness is needed for vulnerable persons like pregnant women, patients with hematological malignancies or organ transplants in particular among first-generation immigrants.

## Background

In the Netherlands, primary varicella infection (chicken pox) is considered a benign common childhood illness. After a first infection with varicella-zoster virus (VZV), immunity is regarded as life-long [1]. In later life, reactivation of latent VZV established in cells of the dorsal root ganglia after primary infection, may cause herpes zoster (shingles). Currently, routine vaccination against VZV is not done, although its introduction in the national immunization programme is now being evaluated [2].To assess the risk of infection after VZV exposure sound epidemiological data are needed to identify risk groups associated with VZV seronegativity.

VZV circulates widely in the Dutch population, and exposure to the virus is relatively frequent. The risk for VZV infection is highest in pre-school children aged 1–5 years, and by the age of 5, at least 93% of the children have VZV antibodies. According to a national population-based VZV seroprevalence study (1995–1996), almost all Dutch adults (98–100%) have antibodies against VZV, yet that study included relatively few residents of non-Dutch origin [3-5]. This finding of near-total VZV seropositivity in the adult Dutch population, and the high risk of infection in young children is typical for a country in a region with a temperate climate. For unclear reasons the epidemiology of VZV shows great regional and seasonal variation. In subtropical and tropical climates the overall incidence of VZV infections is lower and infection occurs often at a later age; physical factors like different levels of ultra-violet radiation may play a role [1,6,7]. As the city of Amsterdam has large immigrant communities originating from various subtropical and tropical countries, such as Morocco, Surinam, and Turkey with probably lower VZV transmission, this study aimed to estimate the seroprevalence of VZV IgG antibodies (anti-VZV) among various ethnic groups in Amsterdam, and identify factors associated with seronegative VZV status.

## Methods

### Study population and sampling procedure

The data used for this study were obtained from a cross-sectional population-based health survey (the Amsterdam Health Monitor, or AHM). The survey was carried out in 2004 by the Amsterdam Public Health Service (GGD) in collaboration with the National Institute for Public Health and the Environment (RIVM). Amsterdam consists of thirteen districts and the sample for the survey was drawn from five of them. These five contain a population that was representative concerning socioeconomic status and ethnicity for the total population of Amsterdam. The survey was approved by the Medical Ethics Committee of the Academic Medical Centre. The sample was stratified by ethnic background (Dutch, Moroccan, Turkish and other ethnic groups) and five age groups (18–34, 35–44, 45–54, 55–64, and 65 years or older). Within each stratum a random sample was drawn. The Turkish and Moroccan ethnic groups were oversampled to ensure sufficient numbers of individuals from these groups. Data were weighted to correct for oversampling by ethnic groups. After weighting the data for age, gender and ethnicity, respondents appeared to have an annual income and unemployment rate that was comparable to that of the total population of Amsterdam in 2004. The overall response rate among ethnic Dutch, Turkish and Moroccan subjects was 45%. More details on the survey are described elsewhere [8,9].

Respondents were invited for an interview and medical examination in a community health centre. All interviews were conducted in the language of choice of the respondent (i.e. Dutch, Turkish, Moroccan-Arabic or Berber). From all the issues that were addressed the following variables were considered pertinent to the current study: sex, age, country of birth of the participant and his/her parents, age at the time of migration, and the number and age of children living with the participant currently and/or in the past. Participants were classified into five ethnic groups (Dutch, Surinamese or Antillean, Turkish, Moroccan, and other), according to the self-reported country of birth and the country of birth of the respondent's mother or father. Furthermore, participants of non-Dutch ethnic origin were classified as first- or second-generation immigrants depending on their own country of birth. Those born in the Netherlands were considered as second-generation and all others as first-generation immigrants.

Participants were requested to provide a blood sample. These were collected, after obtaining written informed consent, and were stored at 7°C within 30 minutes, then centrifuged and frozen at −80°C within 48 hr. Seventy-nine per cent of the participants (n = 1,376) donated a blood sample for the serum repository.

### Serological assays

Plasma samples were tested for IgG-class antibodies to VZV by means of quantitative enzyme immunoassays. The assays were performed in the Public Health Laboratory in Amsterdam according to the instructions of the manufacturer. The serological test was a microplate enzyme-linked immunosorbent assay system that uses purified antigen (cell lysate of a human fibroblast cell line, VZV wild strain) to detect VZV IgG (EUROIMMUN Anti-Varicella-Zoster-Virus IgG-ELISA; Medizinische Labordiagnostika AG, Lübeck, Germany).

In estimating anti-VZV seroprevalence, only samples with a positive result were considered immune. All negative and equivocal test results (according to the manufacturer all results between 80 and 110 mIU/ml) were considered as not immune. We thereby increased the specificity and reduced the number of false positive results. As the purpose of this study was to establish factors associated with VZV susceptibility, reduced assay sensitivity is preferable to reduced specificity [10].

### Statistical analysis

In order to obtain results representative for the adult population in Amsterdam, prevalences and confidence intervals of 95% (95%CI) corrected for stratification were calculated using the complex samples modules of SPSS, version 17 (SPSS Inc., Chicago, Illinois, USA). In these analyses data were weighted for age, sex and ethnic origin, using a weighing method which corrected the oversampling by ethnic groups as described elsewhere [8,11,12]. Prevalences (P) and odds ratios (OR) were estimated in the general Amsterdam population by taking into account the study design using Intercooled Stata 11.1 for Windows (Stata Corp., College Station, Texas, USA). Prevalences were compared using the Chi-square test; and a P-value of <0.05 was considered as significant.

## Results

### Characteristics of the study sample

For this study, 97.5% (1,341/1,376) of the collected blood samples were available for laboratory analysis. Table 1 shows the characteristics of the 1,341 participants who were included. The table shows both the distribution in the study sample (non-weighted), and the estimated distribution representing the Amsterdam population. The study sample consisted of 619 men (46%) and 717 women (54%). Age ranged from 17 to 90 years. The median age for men was 52 years (interquartile range (IQR) 41–62 years) and for women 47 years (IQR 37–58 years). Most participants were of Dutch (33%), Turkish (24%), or Moroccan (21%) ethnic origin. Within ethnic groups, there was an unequal distribution between the sexes for participants of Surinamese and Antillean ethnic origin (71% was female), for participants of Dutch ethnic origin (59% was female), and for participants of Moroccan ethnic origin (57% was male). Of all 1,341 participants 61% (814) were born outside the Netherlands, of which the majority was first-generation Turkish (306) or Moroccan immigrant (262). Other first-generation immigrants came often from the Republic of Surinam and the Netherlands Antilles (78) or from Indonesia (32). The median age at immigration was 25 years (range 0–76 years), and only a minority (64 or 8%) of the immigrants came to live in the Netherlands before the age of 11 years. Only 4% (59) were second-generation immigrants. Half of the participants (47%) had one or more children (median 2 children, range 1–10 children), and 29% none. For 23% of the group, data on having children were missing.

**Table 1:** Characteristics of 1,341 participants of the Amsterdam Health Monitoring Survey, 2004

**Characteristics**	**Study sample**		**Amsterdam adult population**
	**n**	**(%)**	**(Estimated proportion)**
Total	1,341		
Sex			
Female	717	(53.5)	50.3
Male	619	(46.2)	49.7
Sex missing	5	(0.4)	-
Age category			
18–34	212	(15.8)	35.1
35–44	291	(21.7)	23.8
45–54	325	(24.2)	17.1
55–64	279	(20.8)	12.0
65 and older	224	(16.7)	12.0
Age missing	10	(0.7)	-
Ethnic origin			
Dutch	437	(32.6)	53.5
Moroccan	275	(20.5)	6.9
1st generation, Moroccan	262	-	-
Surinamese or Antillean	88	(6.6)	9.3
1st generation, Surinamese or Antillean	78	-	-
Turkish	319	(23.8)	4.4
1st generation, Turkish	306	-	-
Other ethnic origin	212	(15.8)	25.9
1st generation, other ethnic origin	189	-	-
Ethnic origin missing	10	(0.7)	-
Immigration status			
Autochthonous (born in the Netherlands)	516	(38.5)	64.4
Immigrated at age ≤ 10 years	64	(4.8)	5.8
Immigrated at age > 10 years	721	(53.8)	29.8
Immigrated at unknown age	32	(2.4)	-
Country of birth missing	8	(0.6)	-
Having children			
Yes	634	(47.3)	55.7
No	394	(29.4)	44.3
Missing	313	(23.3)	-
Number of children (*n = 1,028*)			
0	394	(38.3)	44.8
1	203	(19.7)	21.6
2	239	(23.2)	22.0
3 or more	176	(17.1)	11.6
Number of children missing	16	(1.2)	-

### Seroprevalence of anti-VZV in the Amsterdam population

Table 2 gives an overview of the test results and the estimated anti-VZV seroprevalence by demographic characteristics. The anti-VZV seroprevalence is shown both non-weighted, representing the study sample, and weighted, representing the Amsterdam population. The anti-VZV seroprevalence in the overall Amsterdam population was estimated to be 93.8% (95% CI 91.6–95.5%). Regarding ethnic origin, seroprevalence was lowest among first-generation immigrants (Moroccan immigrants 90%, Surinamese or Antillean immigrants 91%, and Turkish 92%) compared to those born in the Netherlands (Dutch ethnic origin and second-generation immigrants 97–98%). Among the first-generation immigrants, those that migrated before the age of 11 were more likely to be seropositive, than those that migrated at a later age (P < 0.001).

**Table 2:** Prevalence of VZV IgG antibodies by demographic characteristics among 1,341 Amsterdam residents aged 18 years and older, 2004

	**Study sample**				**The overall Amsterdam population**	
	**n**	**% negative**	**% equivocal**	**% positive**	**% anti-VZV seropositive (95% CI)**	**P-value**
Total	1,341	4.1	2.8	93.1	93.8 (91.6–95.5)	
Sex						0.29
Male	619	4.2	2.9	92.9	92.8 (89.0–95.4)	
Female	717	4.0	2.8	93.2	94.8 (92.2–96.6)	
Age category						0.42
18–34 years	212	4.7	2.4	92.9	92.0 (86.3–95.5)	
35–44 years	291	4.1	3.4	92.4	95.0 (90.8–97.3)	
45–54 years	325	4.9	3.1	92.0	93.5 (89.3–96.1)	
55–64 years	279	2.2	2.5	95.3	96.4 (92.4–98.4)	
65 years and older	224	4.5	2.7	92.9	94.8 (89.9–97.4)	
Age missing	10	10.0	0	90.0	-	
Ethnic origin						0.03
Dutch	437	2.1	1.8	96.1	96.6 (94.3–98.0)	
Surinamese and Antillean	89	5.6	1.1	93.3	93.0 (80.2–97.8)	
Turkish	319	4.7	4.7	90.6	91.8 (87.1–94.8)	
Moroccan	275	5.8	3.6	90.6	89.7 (82.9–94.0)	
Other	211	4.7	1.9	93.6	90.0 (82.8–94.3)	
Ethnic origin missing	10	0	0	100	-	
Generation						
Dutch	437	2.1	1.8	96.1	96.6 (94.3–98.0)	0.002
1^st^ generation. Surinamese or Antillean	78	6.4	1.3	92.3	90.6 (74.7–96.9)	
1^st^ generation. Turkish	306	4.6	4.9	90.5	91.5 (86.5–94.7)	
1^st^ generation. Moroccan	262	5.7	3.8	90.5	90.1 (83.5–94.2)	
Other 1^st^ generation	189	5.3	2.1	92.6	87.9 (79.7–93.0)	
2^nd^ generation immigrants	59	3.4	0	96.6	98.3 (91.3–99.7)	
Ethnic origin missing	10	0	0	100	-	
Immigration status						0.02
Autochthonous (born in the Netherlands)	516	2.1	1.6	96.3	-	
Immigrated at age > 10 years	721	5.7	4.0	90.3	86.3 (80.4–90.7)	
Immigrated at age ≤ 10 years	64	1.6	1.6	96.9	97.5 (87.8–99.5)	
Immigrated at unknown age	32	6.3	0	93.8	-	
Country of birth missing	8	0	0	100	-	
Children						0.80
No	394	4.1	2.8	93.2	93.3 (88.7–96.1)	
Yes	634	4.3	3.3	92.4	94.7 (91.1–96.9)	
Data on having children missing	313	3.8	1.9	94.3	-	
Number of children *(n =1,028)*						0.94
0	394	4.1	2.8	93.2	93.3 (88.7–96.1)	
1	203	4.4	2.0	93.6	94.9 (88.2–97.9)	
2	239	5.0	3.8	91.2	94.7 (87.5–97.8)	
3 or more	176	3.4	4.5	92.1	93.6 (84.2–97.5)	
Number of children missing	16	0	0	100	-	

### Predictors for IgG seronegativity to VZV in the study sample

The results of the univariable analysis for IgG seronegativity to VZV are shown in Table 3. All negative and equivocal test results were considered as IgG seronegative. Both ethnic origin and generation of immigration were positive predictors for IgG seronegativity to VZV. When considering the ethnic origin, people of Moroccan and Turkish ethnic origin were 2.5 times more likely to be seronegative compared to people from Dutch ethnic origin (P = 0.013). Almost 10% of all first-generation immigrants from Morocco and Turkey were anti-VZV seronegative (P = 0.015). Immigrants that migrated after the age of 10 were more likely to be seronegative compared to those that migrated at a younger age and those born in the Netherlands (P = 0.0001). No other predictors for seronegativity were found.

**Table 3:** IgG seronegativity to VZV by demographic characteristics in the study sample aged 18 years and older, 2004

	**n**	**VZV seronegative**		**Univariable OR (95%)**	**P-value**
		**n**	**%**		
Total	1,341	93	6.9 (5.6–8.4)		
Sex					0.84
Male	619	44	7.1	1	
Female	717	49	6.8	0.96 (0.6–1.5)	
Age category					0.52
18–34 years	212	15	7.1	1	
35–44 years	291	22	7.6	1.07 (0.5–2.1)	
45–54 years	325	26	8.0	1.14 (0.6–2.2)	
55–64 years	279	13	4.7	0.64 (0.3–1.4)	
65 years and older	224	16	7.1	1.01 (0.5–2.1)	
Age missing	10	1	10.0	-	
Ethnic origin					0.013
Dutch	437	17	3.9	1	
Surinamese or Antillean	89	6	6.7	1.79 (0.7–4.7)	
Turkish	319	30	9.4	2.56 (1.4–4.7)	
Moroccan	275	26	9.5	2.58 (1.4–4.8)	
Other	211	14	6.6	1.76 (0.8–3.6)	
Ethnic origin missing	10	0	0	-	
Generation					
Dutch	437	17	3.9	1	
1^st^ generation, Surinamese or Antillean	78	6	7.7	2.06 (0.8–5.4)	
1^st^ generation, Turkish	306	29	9.5	2.59 (1.4–4.8)	
1^st^ generation, Moroccan	262	25	9.5	2.61 (1.4–4.9)	
Other 1^st^ generation	189	14	7.4	1.98 (1.0–4.1)	
2^nd^ generation immigrants	59	2	3.4	0.87 (0.2–3.9)	
Ethnic origin missing	10	0	0	-	
Immigration status					< 0.0001
Autochthonous (born in the Netherlands)	516	19	3.7	0.36 (0.2–0.6)	
Immigrated at age > 10 years	721	70	9.7	1	
Immigrated at age ≤ 10 years	64	2	3.1	0.3 (0.1–1.3)	
Immigrated at unknown age	32	2	6.3	-	
Country of birth missing	8	0	0	-	
Children					0.57
No	394	27	6.9	1	
Yes	634	48	7.6	1.11 (0.7–1.8)	
Data on having children missing	313	18	5.8	0.83 (0.5–1.5)	
Number of children *(n =1,028)*					0.75
0	394	27	6.9	1	
1	203	13	6.4	0.93 (0.5–1.8)	
2	239	21	8.9	1.31 (0.7–2.4)	
3 or more	176	14	8.0	1.17 (0.6–2.3)	
Number of children missing	16	0	0	-	

## Discussion

Our study shows a high seroprevalence (94%) of VZV IgG antibodies in the overall adult Amsterdam population (95%CI 92–96%), which is in line with other seroprevalence estimates in adults living in temperate zones [3,5,13-19]. A comparative sero-epidemiology study of anti-VZV in 11 countries in the European region found that seroprevalence was above 90% in all countries, except for Italy (88.8%)[5] . The estimated seroprevalence in Amsterdam is rather low, compared to the near-total VZV seropositivity (97–100%) in the adult Dutch population, but probably representative for a highly urbanized area. In a national population-based seroprevalence study in 1995, a significantly lower seroprevalence (93.6%; 95%CI 91.7–95.8%) was found in highly urbanized municipalities, compared to rural regions (95.9%; 95%CI 95.2–96.6%) [Personal communication; H. de Melker, Data from 'PIENTER 1995–1996']. Urbanization of < 2500 addresses per square kilometer (sq.km) was an independent predictor for seropositivity of VZV compared to urbanization ≥2500 addresses per sq. km (OR 2.1; 95% CI 1.1–3.7) [3]. The difference was not easily explained [3,5,13,16,20,21].

One explanation for this difference may be the ethnic diversity present in urban populations. The city of Amsterdam has large migrant communities, with people originating from various subtropical and tropical countries, which are known to have less VZV transmission. In temperate regions, VZV causes annual epidemics among susceptible household members, in day care centers, and in schools, resulting in high seroprevalence. In warmer climates, VZV infection is less frequent and as many as 50% of young adults in tropical countries may never have had a primary VZV infection [1,5,7,22]. In this study, the relatively low anti-VZV seroprevalence in the Amsterdam adult population is explained by the presence of susceptible immigrants. First-generation immigrants did have a significantly lower seroprevalence than persons who were born in the Netherlands. On average, first-generation immigrants had a 2 times higher risk of being anti-VZV seronegative. Furthermore there was a positive association between anti-VZV seroprevalence and the age of migration. In this study, the median age at immigration was 25 years, and most participating immigrants (665 or 84%) migrated more than 11 years ago (data not shown). Immigrants who migrated after the age of 11 years were more likely to be seronegative compared to those that immigrated at a younger age. It is likely that new immigrants, especially the children, experience VZV infection after settling in the Netherlands, yet data on the incidence of VZV in immigrants in the Netherlands are lacking. A good number of Surinamese people who migrated to the Netherlands after the independence of Surinam in 1975, were referred to the outpatient department for sexually transmitted infections because of a vesicular rash, which was thought to be secondary syphilis but turned out to be chickenpox [Personal communication; A, van den Hoek]. Also, an outbreak of chickenpox among West-Indians residing in the Netherlands has been described [23]. Several surveys in other countries describe a low seroprevalence in immigrants and outbreaks of chickenpox among newly arrived migrants [24-27]. As in this study the only three variables eligible for inclusion into a multivariable model (ethnic origin; ethnic origin & generation; immigration status) were nearly identical (‘ethnic origin and ethnic origin & generation’) or collinear (‘immigration status’ and ‘ethnic origin’), a multivariable analysis was not feasible.

Other studies have described that anti-VZV seroprevalence may be related to household composition (≥ 4 persons) and school attendance by a household member [3,6,28-30] . However, in this study no association between anti-VZV seroprevalence and having children or the number of children was found.

In this study, we increased the specificity of the test by considering those with equivocal test results as non-immune. This may have led to an underestimate of the true anti-VZV seroprevalence and thus the immunity in the Amsterdam population, and overestimated the VZV susceptibility. However, as the aim of this study was to identify factors associated with VZV susceptibility, and in order to reduce the number of false positives, this approach seems justified. A subsidiary analysis in which the equivocal test results were considered as false negative showed similar, although less significant outcomes (data not shown).

The relatively low response rate of the AHM (45%) and its sampling methods may be considered as potential sources of bias, which may have affected the results of this study. However, the oversampling and non-response bias by ethnic groups were addressed by weighing the data by sex, age, and ethnicity. A non-responders survey showed that the sample appeared to be representative of the population on most health determinants [9]. Furthermore, a direct association between VZV infection and response to the AHM seems unlikely. For these reasons the weighted VZV prevalence may be considered representative for the whole adult population of Amsterdam.

The introduction of a two-dose universal childhood VZV vaccination programme in the Netherlands is being considered. In terms of health policies and the cost-effectiveness of the introduction of a universal vaccination programme for VZV, the finding of 4–8% of adult susceptibles in Amsterdam should be taken into account. One of several unresolved questions is the impact of a VZV vaccination programme on the incidence of herpes zoster. Another issue related to cost-effectiveness is the uncertainty of the burden of disease of VZV in children [2]. Compared to neighboring countries, the Netherlands reports lower rates of complications of chickenpox in children [31]. With a universal childhood programme a shift in the age of primary VZV infection from childhood to adolescents and adults is likely to occur [5,32-35]. Primary VZV in adults and adolescents have, like pregnant women and immune-compromised individuals, an increased risk of complications [1,29,36,37]. As in the Netherlands chickenpox is not a notifiable disease, little is known on the incidence of primary VZV infection or its complications in adults, and the current overall burden of VZV infection in the adult population cannot be estimated. Improved surveillance is needed as a universal childhood vaccination programme will only change the risk of infection in VZV-negative adults long after its introduction.

## Conclusion

In conclusion, the results of this study imply that about 4-8% of the general adult Amsterdam population is still susceptible to infection with VZV, and that susceptibility is even higher in some immigrant groups. When assessing the risk of infection after VZV exposure alertness is needed for vulnerable persons like pregnant women, patients with hematological malignancies or organ transplants in particular among first-generation immigrants. [38].

## Competing interests

The authors declare that they have no competing interests.

## Authors’ contributions

GGCvR performed the data analysis and wrote the first draft of the manuscript. MD advised and supervised the carrying out of the immunoassays. MSvdL contributed to the statistical analysis. GBS and AvdH made substantial changes to the manuscript. All authors read and approved the final manuscript.

## Pre-publication history

The pre-publication history for this paper can be accessed here:

http://www.biomedcentral.com/1471-2334/12/140/prepub

## References

[B1] Arvin AM Varicella-zoster virus Clin Microbiol Rev 1996 9 361 381 8809466 10.1128/cmr.9.3.361 PMC172899

[B2] The future of the National Immunisation Programme: towards a programme for all age groups 2007 http://www.gezondheidsraad.nl/en/publications/future-national-immunisation-programme-towards-programme-all-age-groups.

[B3] de Melker HE Berbers G Hahne S Rumke H van den Hof S de Wit A Boot H The epidemiology of varicella and herpes zoster in The Netherlands: implications for varicella zoster virus vaccination Vaccine 2006 24 3946 3952 10.1016/j.vaccine.2006.02.017 16564115

[B4] de Melker HE Nagelkerde NJ Conyn-van Spaendonck MA Non-participation in a population-based seroprevalence study of vaccine-preventable diseases Epidemiol Infect 2000 124 255 262 10.1017/S0950268899003234 10813151 PMC2810909

[B5] Nardone A de Ory F Carton M Cohen D van Damme P Davidkin I Rota MC de Melker H Mossong J Slacikova M The comparative sero-epidemiology of varicella zoster virus in 11 countries in the European region Vaccine 2007 25 7866 7872 10.1016/j.vaccine.2007.07.036 17919788

[B6] Nichols RA Averbeck KT Poulsen AG al Bassam MM Cabral F Aaby P Breuer J Household size is critical to varicella-zoster virus transmission in the tropics despite lower viral infectivity Epidemics 2011 3 12 18 10.1016/j.epidem.2010.11.003 21420656 PMC3072572

[B7] Rice PS Ultra-violet radiation is responsible for the differences in global epidemiology of chickenpox and the evolution of varicella-zoster virus as man migrated out of Africa Virol J 2011 8 189 10.1186/1743-422X-8-189 21513563 PMC3094303

[B8] Agyemang C Ujcic-Voortman J Uitenbroek D Foets M Droomers M Prevalence and management of hypertension among Turkish, Moroccan and native Dutch ethnic groups in Amsterdam, the Netherlands: The Amsterdam Health Monitor Survey J Hypertens 2006 24 2169 2176 10.1097/01.hjh.0000249693.73618.c9 17053537

[B9] Uitenbroek DG Ujcic-Voortman JK Janssen AP Tichelman PJ Verhoeff AP Gezond zijn en geoznd leven in Amsterdam 2006 the Amsterdam Health Monitor, Amsterdam http://www.os.amsterdam.nl/pdf/2006_gezondheidsmonitor_2004.pdf

[B10] Breuer J Schmid DS Gershon AA Use and limitations of varicella-zoster virus-specific serological testing to evaluate breakthrough disease in vaccinees and to screen for susceptibility to varicella J Infect Dis 2008 197 Suppl 2 S147 S151 18419389 10.1086/529448

[B11] Baaten GG Sonder GJ Dukers NH Coutinho RA Van den Hoek JA Population-based study on the seroprevalence of hepatitis A, B, and C virus infection in Amsterdam, 2004 J Med Virol 2007 79 1802 1810 10.1002/jmv.21009 17935187

[B12] Fassaert T de Wit MA Verhoeff AP Tuinebreijer WC Gorissen WH Beekman AT Dekker J Uptake of health services for common mental disorders by first-generation Turkish and Moroccan migrants in the Netherlands BMC Public Health 2009 9 307 10.1186/1471-2458-9-307 19698174 PMC2737538

[B13] Heininger U Braun-Fahrlander C Desgrandchamps D Glaus J Grize L Wutzler P Schaad UB Seroprevalence of varicella-zoster virus immunoglobulin G antibodies in Swiss adolescents and risk factor analysis for seronegativity Pediatr Infect Dis J 2001 20 775 778 10.1097/00006454-200108000-00011 11734740

[B14] Khoshnood B Debruyne M Lancon F Emery C Fagnani F Durand I Floret D Seroprevalence of varicella in the French population Pediatr Infect Dis J 2006 25 41 44 10.1097/01.inf.0000195636.43584.bb 16395101

[B15] Mossong J Putz L Schneider F Seroprevalence and force of infection of varicella-zoster virus in Luxembourg Epidemiol Infect 2004 132 1121 1127 10.1017/S0950268804002754 15635970 PMC2870204

[B16] Salleras L Dominguez A Vidal J Plans P Salleras M Taberner JL Seroepidemiology of varicella-zoster virus infection in Catalonia (Spain). Rationale for universal vaccination programmes Vaccine 2000 19 183 188 10.1016/S0264-410X(00)00178-X 10930671

[B17] Thiry N Beutels P Shkedy Z Vranckx R Vandermeulen C Wielen MV Damme PV The seroepidemiology of primary varicella-zoster virus infection in Flanders (Belgium) Eur J Pediatr 2002 161 588 593 10.1007/s00431-002-1053-2 12424583

[B18] Vyse AJ Gay NJ Hesketh LM Morgan-Capner P Miller E Seroprevalence of antibody to varicella zoster virus in England and Wales in children and young adults Epidemiol Infect 2004 132 1129 1134 10.1017/S0950268804003140 15635971 PMC2870205

[B19] Wutzler P Farber I Wagenpfeil S Bisanz H Tischer A Seroprevalence of varicella-zoster virus in the German population Vaccine 2001 20 121 124 10.1016/S0264-410X(01)00276-6 11567755

[B20] Liyanage NP Fernando S Malavige GN Mallikahewa R Sivayogan S Jiffry MT Vitarana T Seroprevalence of varicella zoster virus infections in Colombo district, Sri Lanka Indian J Med Sci 2007 61 128 134 10.4103/0019-5359.30747 17337813

[B21] Mandal BK Mukherjee PP Murphy C Mukherjee R Naik T Adult susceptibility to varicella in the tropics is a rural phenomenon due to the lack of previous exposure J Infect Dis 1998 178 Suppl 1 S52 S54 9852974 10.1086/514262

[B22] Knowles SJ Grundy K Cahill I Cafferkey MT Susceptibility to infectious rash illness in pregnant women from diverse geographical regions Commun Dis Public Health 2004 7 344 348 15779804

[B23] Huisman J An outbreak of varicella among a group of West Indians residing in the Netherlands Ned Tijdschr Geneeskd 1966 110 2099 2101 5978899

[B24] Barnett ED Christiansen D Figueira M Seroprevalence of measles, rubella, and varicella in refugees Clin Infect Dis 2002 35 403 408 10.1086/341772 12145723

[B25] Gabutti G Fedele A Aprile V Guido M Lopalco P Immigration flows and new epidemiological evidence in southern Italy Vaccine 2003 21 399 400 10.1016/S0264-410X(02)00402-4 12531638

[B26] Kjersem H Jepsen S Varicella among immigrants from the tropics, a health problem Scand J Soc Med 1990 18 171 174 2237323 10.1177/140349489001800303

[B27] Merrett P Schwartzman K Rivest P Greenaway C Strategies to prevent varicella among newly arrived adult immigrants and refugees: a cost-effectiveness analysis Clin Infect Dis 2007 44 1040 1048 10.1086/512673 17366446

[B28] Cohen DI Davidovici BB Smetana Z Balicer RD Klement E Mendelson E Green MS Seroepidemiology of Varicella zoster in Israel prior to large-scale use of varicella vaccines Infection 2006 34 208 213 10.1007/s15010-006-6604-4 16896579

[B29] Heininger U Seward JF Varicella Lancet 2006 368 1365 1376 10.1016/S0140-6736(06)69561-5 17046469

[B30] Silhol R Alvarez FP Arena C Amoros JP Flahault A Hanslik T Boelle PY Micro and macro population effects in disease transmission: the case of varicella Epidemiol Infect 2010 138 482 490 10.1017/S0950268809990896 19796448

[B31] van Lier A van der Maas NA Rodenburg GD Sanders EA de Melker HE Hospitalization due to varicella in the Netherlands BMC Infect Dis 2011 11 85 10.1186/1471-2334-11-85 21466668 PMC3080814

[B32] Brisson M Edmunds WJ Gay NJ Law B De SG Modelling the impact of immunization on the epidemiology of varicella zoster virus Epidemiol Infect 2000 125 651 669 10.1017/S0950268800004714 11218215 PMC2869648

[B33] Boot HJ de Melker HE Stolk EA de Wit GA Kimman TG Assessing the introduction of universal varicella vaccination in the Netherlands Vaccine 2006 24 6288 6299 10.1016/j.vaccine.2006.05.071 16790302

[B34] Brisson M Edmunds WJ Gay NJ Varicella vaccination: impact of vaccine efficacy on the epidemiology of VZV J Med Virol 2003 70 Suppl 1 S31 S37 12627484 10.1002/jmv.10317

[B35] Sengupta N Booy R Schmitt HJ Peltola H Van-Damme P Schumacher RF Campins M Rodrigo C Heikkinen T Seward J Varicella vaccination in Europe: are we ready for a universal childhood programme? Eur J Pediatr 2008 167 47 55 17334784 10.1007/s00431-007-0424-0

[B36] Boelle PY Hanslik T Varicella in non-immune persons: incidence, hospitalization and mortality rates Epidemiol Infect 2002 129 599 606 10.1017/S0950268802007720 12558344 PMC2869923

[B37] Noorda J Hoebe CJ Fatal outbreak of chickenpox (varicella-zoster virus infection) among institutionalised adults with learning difficulties Commun Dis Public Health 2004 7 164 168 15481205

[B38] Leikin E Figueroa R Bertkau A Lysikiewicz A Visintainer P Tejani N Seronegativity to varicella-zoster virus in a tertiary care obstetric population Obstet Gynecol 1997 90 511 513 10.1016/S0029-7844(97)00353-0 9380306

